# Phase Evolution and Structure–Property Relationships of Fe_79_Zr_6_Si_14_Cu_1_ Coatings with Low GFA Produced by Atmospheric Plasma Spraying

**DOI:** 10.3390/ma19214573

**Published:** 2026-10-29

**Authors:** Inez Kredowska, Wirginia Pilarczyk, Aleksandra Małachowska, Paweł Sokołowski, Katarzyna Młynarek-Żak, Paweł M. Nuckowski

**Affiliations:** 1 Faculty of Mechanical Engineering, Silesian University of Technology, Ul. Akademicka 2A, 44-100 Gliwice, Poland; wirginia.pilarczyk@polsl.pl (W.P.); katarzyna.mlynarek-zak@polsl.pl (K.M.-Ż.); pawel.nuckowski@polsl.pl (P.M.N.); 2 Faculty of Mechanical Engineering, Wrocław University of Science and Technology, Wyb. Wyspiańskiego 27, 50-370 Wrocław, Poland; aleksandra.malachowska@pwr.edu.pl (A.M.); pawel.sokolowski@pwr.edu.pl (P.S.)

**Keywords:** atmospheric plasma spraying, Fe-based alloy coatings, low glass-forming ability, microstructure–property relationships, tribological behaviour, corrosion resistance

## Abstract

The phase evolution of low-glass-forming-ability (low-GFA) Fe-Zr-Si alloys under atmospheric plasma spraying (APS) conditions remains insufficiently understood. In the present study, Fe_79_Zr_6_Si_14_Cu_1_ coatings were deposited by APS in order to investigate the influence of spray distance on microstructural development and structure–property relationships. Despite the rapid solidification conditions associated with APS, all coatings exhibited predominantly crystalline multiphase microstructures composed of α-Fe(Si) and Fe-Zr intermetallic phases, with no detectable amorphous fraction in the XRD patterns. The coatings developed a characteristic lamellar morphology with porosity ranging from 2.2 to 4.9%, strongly dependent on spray distance and particle thermal history. Increasing spray distance promoted higher hardness and improved wear resistance but simultaneously deteriorated corrosion resistance due to increased porosity and interlamellar defect population. Among the investigated conditions, the coating deposited at 90 mm exhibited the most favourable balance of hardness, wear resistance, and electrochemical behaviour owing to its relatively dense and cohesive microstructure. The results demonstrate that phase evolution in low-GFA Fe-Zr-Si-Cu alloys processed by APS is governed not only by rapid cooling rates but also by splat-based solidification, repeated thermal cycling, and heterogeneous nucleation during coating build-up.

## 1. Introduction

Metallic glasses have attracted significant scientific and technological interest due to their unique combination of mechanical, magnetic, and corrosion-related properties. Their formation requires suppression of crystal nucleation and growth during rapid solidification and strongly depends on the glass-forming ability (GFA) of the alloy system [1,2].

Among metallic glass systems, Fe-based alloys are particularly attractive due to their combination of mechanical, magnetic, and corrosion-related properties together with their relatively low material cost compared with noble-metal-based alloys [3,4,5]. The glass-forming ability of Fe-based alloys is highly composition-dependent, and the formation of amorphous structures is commonly facilitated by metalloid additions such as boron, carbon, or phosphorus, which suppress crystallization during rapid solidification. In contrast, boron-free Fe-Zr-Si-based alloys exhibit significantly lower GFA and therefore a stronger tendency toward crystalline phase formation [6].

Previous studies on Fe-Zr-Si alloys focused primarily on melt-spun ribbons [6,7,8,9] and, more recently, on additive manufacturing approaches such as laser powder bed fusion [10]. These investigations demonstrated the formation of ultrafine microstructures together with promising soft magnetic properties. In particular, Cu additions were shown to influence nucleation behaviour and microstructural refinement during rapid solidification [7,8,10]. Nevertheless, the phase evolution of Fe-Zr-Si-Cu alloys under non-equilibrium processing conditions remains insufficiently understood, especially in processes involving repeated thermal cycling, localized reheating, and heterogeneous solidification environments.

Atmospheric plasma spraying (APS) is widely used for the deposition of metallic and ceramic coatings due to its ability to process a broad range of feedstock materials under high-temperature conditions [11]. During the process, powder particles are heated and accelerated within the plasma jet toward the substrate, where they undergo rapid flattening and solidification upon impact, forming a characteristic lamellar coating structure. Unlike conventional rapid solidification techniques such as melt spinning or additive manufacturing, APS involves successive deposition of individual molten droplets accompanied by repeated thermal cycling, localized reheating, and heterogeneous solidification conditions [12].

As a consequence, phase formation during APS is governed not only by high cooling rates but also by splat-based solidification mechanisms, interlamellar interactions, and localized variations in particle thermal history [13]. These factors strongly influence crystallization behaviour, defect formation, and the resulting functional properties of the deposited coatings, indicating that amorphous phase formation in plasma-sprayed alloys cannot be assumed solely on the basis of rapid cooling conditions.

This study investigates the phase evolution and structure–property relationships of Fe_79_Zr_6_Si_14_Cu_1_ coatings produced by atmospheric plasma spraying, with particular attention devoted to the influence of spray distance on crystallization behaviour, splat-based microstructural development, and the resulting mechanical, tribological, and corrosion properties. To date, the phase evolution of low-GFA Fe-Zr-Si-Cu alloys processed by APS has not been systematically investigated. The present work therefore aims to clarify whether the rapid solidification conditions associated with APS are sufficient to retain amorphous phases in low-GFA Fe-based alloys.

## 2. Materials and Methods

### 2.1. Material

Fe_79_Zr_6_Si_14_Cu_1_ alloy powder (at.%) was used as the feedstock material for coating deposition. The alloy was produced from high-purity elemental constituents, including Fe (>99.9%), Zr (>99.9%), Si (>99.9%) and Cu (>99.9%). Powder fabrication was carried out by gas atomization (Nanoval GmbH & Co. KG, Berlin, Germany), followed by sieving to obtain a particle size fraction of +15/−50 μm. The nominal chemical composition provided by the manufacturer is presented in Table 1.

**Figure 1:**
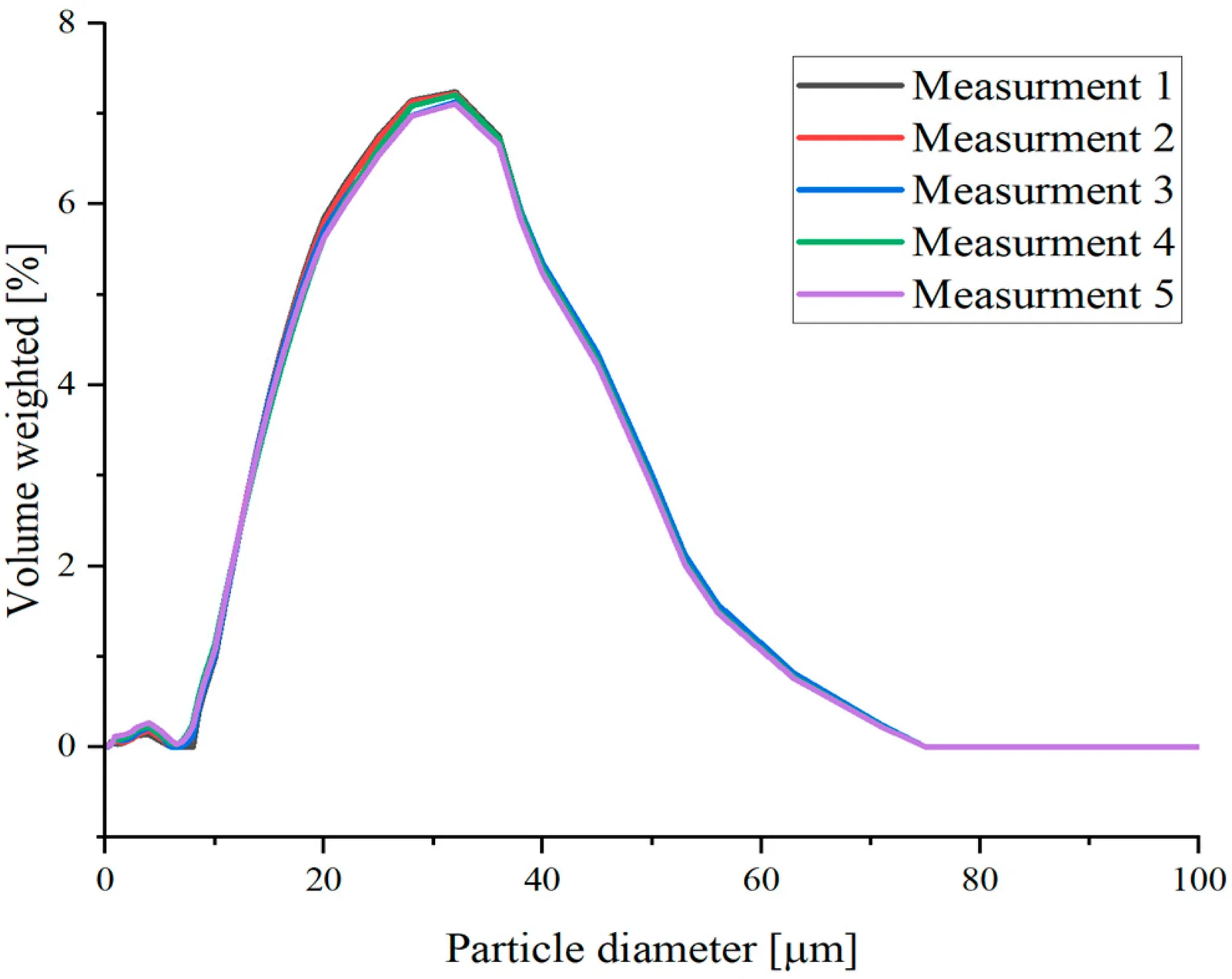
Particle size distribution for five measurements.

Particle size distribution analysis was performed using a PSA 1190 analyser (Anton Paar, Graz, Austria). Five independent measurements were conducted, yielding particle size distribution values of D10 = 12.19 μm, D50 = 24.71 μm, and D90 = 42.55 μm, which are suitable for atmospheric plasma spraying applications. The obtained particle size distribution curves showed good repeatability and similar distribution profiles. The powder exhibited a relatively broad particle size distribution, with the highest volume fraction observed in the range of approximately 25–40 μm. The particle size distribution curves are presented in Figure 1.

### 2.2. Substrate Preparation

AISI 304 stainless steel was used as the substrate material. The substrates were prepared in the form of coupons with dimensions of Ø25 × 3 mm. Prior to coating deposition, the substrate surfaces were grit-blasted using EK-F36 alumina abrasive in order to increase surface roughness and improve coating adhesion. The specimens were subsequently cleaned with ethanol to remove residual contaminants. Before spraying, the feedstock powder was dried to reduce the influence of residual moisture during deposition.

### 2.3. Coating Deposition

Coatings were deposited by atmospheric plasma spraying (APS) using an SG-100 plasma torch (Praxair, Indianapolis, IN, USA) equipped with radial powder injection. The torch was mounted on a six-axis Fanuc R-2000 robotic manipulator (Fanuc, Oshinomura, Japan). To limit excessive thermal input, a relatively low plasma power was applied. Spray distance (SD) was selected as the main variable process parameter, and coatings were deposited at distances of 80, 90, and 100 mm. All samples were mounted on a carousel accommodating 24 samples and sprayed at once, which allowed to keep the same deposition conditions. The remaining deposition parameters are summarized in Table 2.

### 2.4. Characterization

Phase identification was performed by X-ray diffraction (XRD) analysis using an X’Pert Pro diffractometer (PANalytical, Almelo, The Netherlands) equipped with Co Kα radiation (λ = 1.78 Å). Diffraction patterns were collected over a 2θ range from 20° to 120° with a step size of 0.05°.

Microstructural characterization was carried out using a Tescan Vega 3 scanning electron microscope (Tescan, Brno, Czech Republic) operating in backscattered electron (BSE) mode. SEM observations focused on coating morphology, porosity, cracks, and interlamellar defects. The analysis was performed at an accelerating voltage of 20 kV and a working distance of 15 mm.

Coating thickness measurements were performed at 20 different locations for each sample and averaged. Porosity was evaluated using SEM cross-sectional images acquired from different coating regions. The images were processed by threshold analysis using ImageJ version 1.54g software, which was also used for coating thickness measurements.

Tribological tests were performed using a tribometer (CSM Instruments, Peseux, Switzerland) operating in a pin-on-disc configuration under rotary motion. An Al_2_O_3_ ball with a diameter of 6 mm was used as the counterbody. Prior to testing, the sample holder, pin, and counterbody were cleaned with isopropyl alcohol and dried to remove surface contaminants. Tribological measurements were performed under ambient laboratory conditions at room temperature (25 °C). Relative humidity was not controlled during the experiments.

The tests were carried out under a normal load of 10 N. The sliding distance was 500 m, and the sliding speed was 5 cm/s. The counterbody moved along a circular wear track with a diameter of 3 mm. A new Al_2_O_3_ ball was used for each measurement to ensure repeatable testing conditions. Three independent tribological tests were performed for each coating variant. Three tribological tests were performed for each coating variant. Following each test, the cross-sectional area of the wear track was measured at three locations distributed approximately 120° apart along the circular track with Taylor-Hobson Surtonic 25 (Taylor Hobson Ltd., Leicester, UK) These measurements were used to characterize the cross-sectional area of each wear track. The mean wear rate and standard deviation were calculated from the results of the three tribological tests.

A lower sliding speed than that commonly used in tribological testing was intentionally selected due to the relatively low wear resistance of the investigated coatings compared with conventional boron-rich Fe-based amorphous coatings. The specific wear rate was calculated according to Equation (1) [14].

(1) $$W_{r}=\frac{V}{F\cdot s}=\frac{A\cdot \pi \cdot d}{F\cdot s}$$

where:

W_r_—specific wear rate [mm^3^/N·m];

V—wear volume [mm^3^];

A—cross-sectional area of the wear track [mm^2^];

d—wear track diameter [mm];

F—applied normal force [N];

s—sliding distance [m].

Vickers microhardness measurements were performed using a FUTURE-TECH FM-ARS 9000 (Future Tech, Tokyo, Japan) microhardness tester. For each sample, ten indentations were made, and the average hardness value together with the standard deviation was calculated. The measurements were conducted under a load of 100 gf with a dwell time of 15 s.

Corrosion behaviour was evaluated by potentiodynamic polarization measurements carried out in a 3.5 wt.% NaCl solution at 25 °C. The electrochemical measurements were performed using an Autolab PGSTAT302N (Metrohm AG, Herisau, Switzerland) potentiostat equipped with a conventional three-electrode corrosion cell. The exposed surface area of the tested samples was 1 cm^2^. A saturated calomel electrode (SCE) was used as the reference electrode, while a platinum rod served as the counter electrode.

Open-circuit potential (OCP) measurements were conducted for 3600 s prior to polarization testing. Potentiodynamic polarization measurements were subsequently performed at a scan rate of 1 mV/s. Electrochemical data acquisition and analysis were performed using NOVA ver.1.11 software. The corrosion potential (E_corr_), corrosion current density (j_corr_), and polarization resistance (R_p_) were determined using the Stern–Geary method according to Equation (2) [15].

(2) $$j_{\mathrm{corr}}=\frac{\left(\beta_{a}\cdot \beta_{c}\right)}{\left[2.303\left(\beta_{a}+\beta_{c}\right)\right]}\cdot \left(\frac{1}{R_{p}}\right)$$

where:

β_a_—anodic Tafel slope;

β_c_—cathodic Tafel slope.

After electrochemical testing, the coating surfaces were re-examined by scanning electron microscopy (SEM) in secondary electron (SE) mode to characterize the morphology of the corrosion-affected surfaces. The observations were performed at an accelerating voltage of 20 kV and a working distance of approximately 7.0 mm.

## 3. Results and Discussion

### 3.1. Powder Characterization

**Figure 2:**
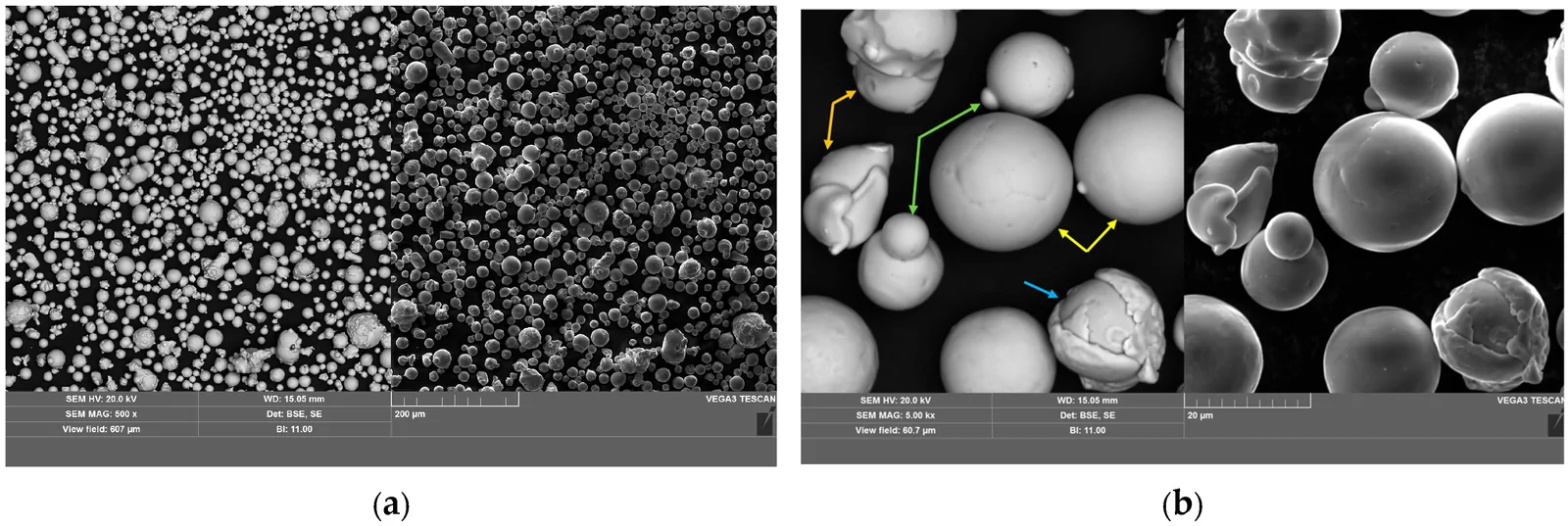
SEM micrographs of gas-atomized Fe_79_Zr_6_Si_14_Cu_1_ powder obtained at different magnifications: (**a**) 500× and (**b**) 5000×.

SEM micrographs of the Fe_79_Zr_6_Si_14_Cu_1_ powder are presented in Figure 2. The powder particles exhibited predominantly spherical morphology characteristic of gas-atomized feedstock materials. The average particle size was 34.3 ± 9.5 μm, which is consistent with the particle size distribution results.

In Figure 2b, the arrows indicate characteristic morphological features observed in the powder particles: yellow—spherical particles; blue—surface cracking; green—satellite particles; and orange—hollow or partially collapsed particles.

Most particles exhibited smooth surfaces, indicating effective melting and rapid solidification during gas atomization. Nevertheless, localized surface irregularities, including wrinkles, fractured regions, and partially collapsed particles, were also observed. In addition, small satellite particles attached to larger particles were detected. Such features are typical for gas-atomized powders and may influence powder flowability and melting behaviour during plasma spraying.

### 3.2. X-Ray Diffraction

**Figure 3:**
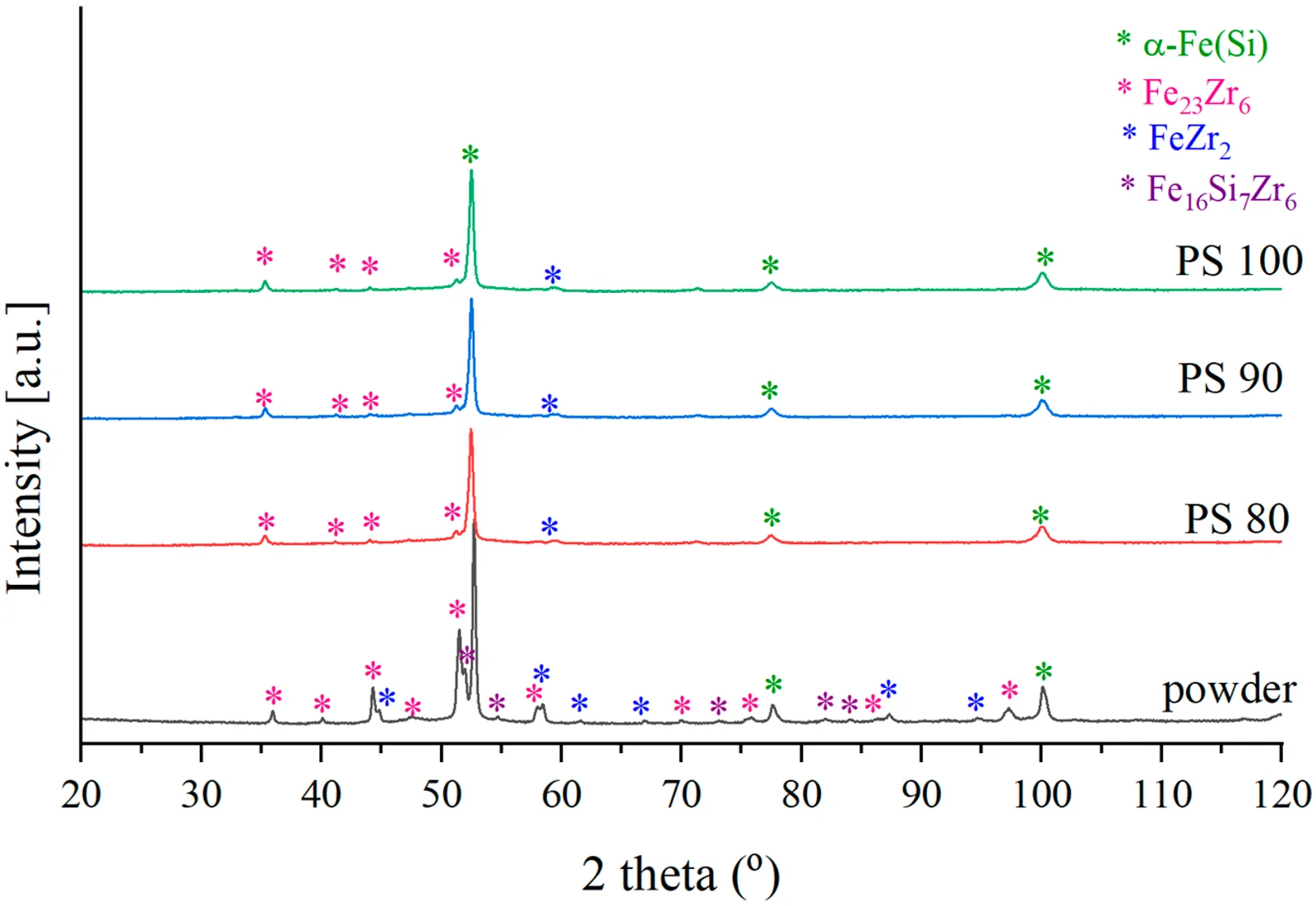
XRD patterns of Fe_79_Zr_6_Si_14_Cu_1_ feedstock powder and coatings deposited by APS at different spray distances.

The X-ray diffraction patterns of the Fe_79_Zr_6_Si_14_Cu_1_ feedstock powder and coatings deposited at different spray distances are presented in Figure 3. The diffraction pattern of the powder exhibited distinct crystalline reflections, indicating that the feedstock material was crystalline prior to the spraying process. The identified reflections could be attributed to α-Fe(Si) and several intermetallic phases, including Fe_23_Zr_6_, FeZr_2_, and the ternary Fe_16_Si_7_Zr_6_ phase. The absence of a broad amorphous halo indicates that the gas-atomized powder did not retain a fully amorphous structure despite the rapid solidification conditions associated with gas atomization. The presence of multiple crystalline phases in the feedstock is consistent with the limited glass-forming ability of the investigated Fe-Zr-Si-Cu alloy.

After atmospheric plasma spraying, all coatings exhibited predominantly crystalline multiphase structures, with no distinct broad amorphous halo detected in the XRD patterns. Compared with the feedstock powder, the Fe_16_Si_7_Zr_6_ phase was no longer detected in the coatings, whereas α-Fe(Si), Fe_23_Zr_6_, and FeZr_2_ were identified. However, the absence of detectable reflections from Fe_16_Si_7_Zr_6_ does not necessarily indicate its complete elimination, as its content may have fallen below the detection limit of the applied XRD technique. Similarly, the absence of a distinct amorphous halo does not exclude the presence of a minor amorphous fraction below the detection limit.

The strongest diffraction peak, at approximately 52° (2θ), resulted from overlapping reflections of α-Fe(Si) and intermetallic phases. The similar diffraction profiles obtained at stand-off distances of 80, 90, and 100 mm indicate that the spraying distance did not substantially affect the qualitative phase constitution within the investigated range. Although variations in particle residence time, in-flight thermal history, and splat solidification may influence phase formation, no additional detectable changes in phase constitution were identified.

Compared with the feedstock powder, the coatings exhibited broadened diffraction peaks. This broadening may be associated with microstructural refinement, including a reduction in crystallite size, and/or lattice strain arising from the thermal history of the particles during APS. However, these contributions cannot be distinguished based on the XRD patterns alone. Despite the rapid cooling conditions associated with APS, the coatings remained predominantly crystalline, and no distinct amorphous fraction was detected by XRD.

Obtained results are consistent with previous studies on Fe-Zr-Si-based alloys with limited glass-forming ability. In [6], Fe_79_Zr_5_Si_15_Cu_1_ alloys produced by melt spinning crystallized into α-Fe(Si) and Fe_23_Zr_6_ phases after annealing, which corresponds well with the phases identified in the present study. Similarly, ref. [7] reported that Fe_80_Zr_5_Si_15_ and Fe_80_Zr_12_Si_8_ alloys exhibited diffraction peaks corresponding primarily to Fe and Fe-Si solid solution phases. The same study demonstrated that Cu addition promoted formation of predominantly amorphous structures in several compositions, confirming that Cu improves the glass-forming ability of Fe-Zr-Si alloys. Nevertheless, the Fe_80_Zr_7_Si_13_ alloy still exhibited a bcc-Fe phase, indicating that Cu addition alone is insufficient to completely suppress crystallization in low-GFA systems. Comparable phase evolution was also reported in [10] for Fe_79_Zr_6_Si_14_Cu_1_ powders and additively manufactured samples containing α-Fe(Si), Fe_23_Zr_6_, FeZr_2_, and Fe_16_Si_7_Zr_6_ phases. Although partial suppression of selected intermetallic phases was achieved by modifying processing conditions, fully amorphous structures were not retained.

The absence of a detectable amorphous fraction in the present coatings may be associated with the specific thermal conditions characteristic of APS processing. Unlike melt spinning, APS involves the successive deposition of molten or partially molten particles, potentially exposing previously deposited splats to additional thermal cycles and localized reheating during coating growth. Such thermal effects may influence the crystallization behaviour of the deposited material. Furthermore, splat boundaries, oxide films, and interlamellar defects may provide potential sites for heterogeneous nucleation, thereby facilitating crystallization under suitable conditions. However, the individual contributions of these mechanisms could not be established from the present results.

A higher amorphous fraction in thermally sprayed coatings has generally been reported for Fe-based alloys containing glass-forming elements such as B, C, and P. Previous studies [16,17] demonstrated that such alloy systems may retain predominantly amorphous structures after plasma spraying, indicating that alloy composition plays an important role in suppressing crystallization under APS conditions.

### 3.3. SEM

**Figure 4:**
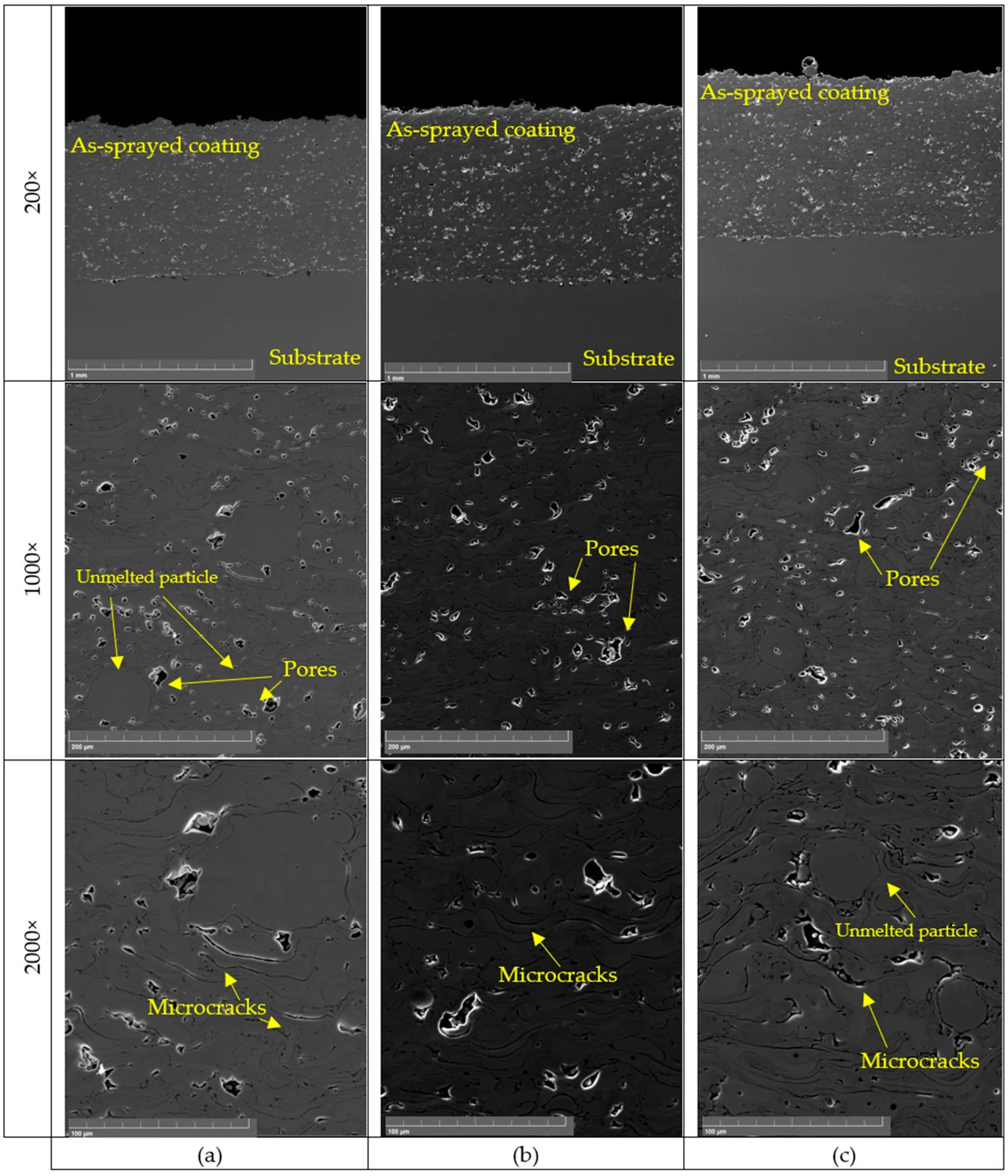
SEM imaging for spraying distances of: (**a**) 80 mm, (**b**) 90 mm, (**c**) 100 mm.

SEM images of the Fe_79_Zr_6_Si_14_Cu_1_ coatings deposited at different spray distances are presented in Figure 4. All coatings exhibited the lamellar microstructure characteristic of atmospheric plasma sprayed materials, formed through successive impact and rapid solidification of molten splats on the substrate surface. Interlamellar boundaries, pores, microcracks, and partially unmelted particles were observed in each coating. The coating/substrate interface remained continuous regardless of spray distance, indicating good mechanical interlocking after grit blasting.

The coating deposited at 80 mm (Figure 4a) reached an average thickness of 614 ± 17 μm and exhibited the lowest porosity of 2.2 ± 0.3%. The relatively short spray distance enabled particles to retain higher thermal energy prior to impact, promoting effective splat deformation and formation of a dense lamellar structure. Nevertheless, isolated pores and partially unmelted particles were still observed.

Increasing the spray distance to 90 mm (Figure 4b) resulted in the thickest coating, with an average thickness of 682.6 ± 16 μm, while maintaining low porosity (2.4 ± 0.4%). Compared with the other coatings, this condition produced a more homogeneous lamellar microstructure with fewer interlamellar discontinuities and improved splat cohesion.

A further increase in spray distance to 100 mm (Figure 4c) was accompanied by changes in the coating microstructure. The coating thickness decreased to 628.4 ± 17 μm, whereas the porosity increased to 4.9 ± 0.4%. More frequent interlamellar voids, partially unmelted particles, and structural discontinuities were observed throughout the coating. These features may be associated with changes in the thermal conditions experienced by the particles during their longer flight through the plasma plume. In particular, particle cooling prior to impact may have affected their melting state and splat spreading behaviour, potentially contributing to the formation of interlamellar defects.

**Figure 5:**
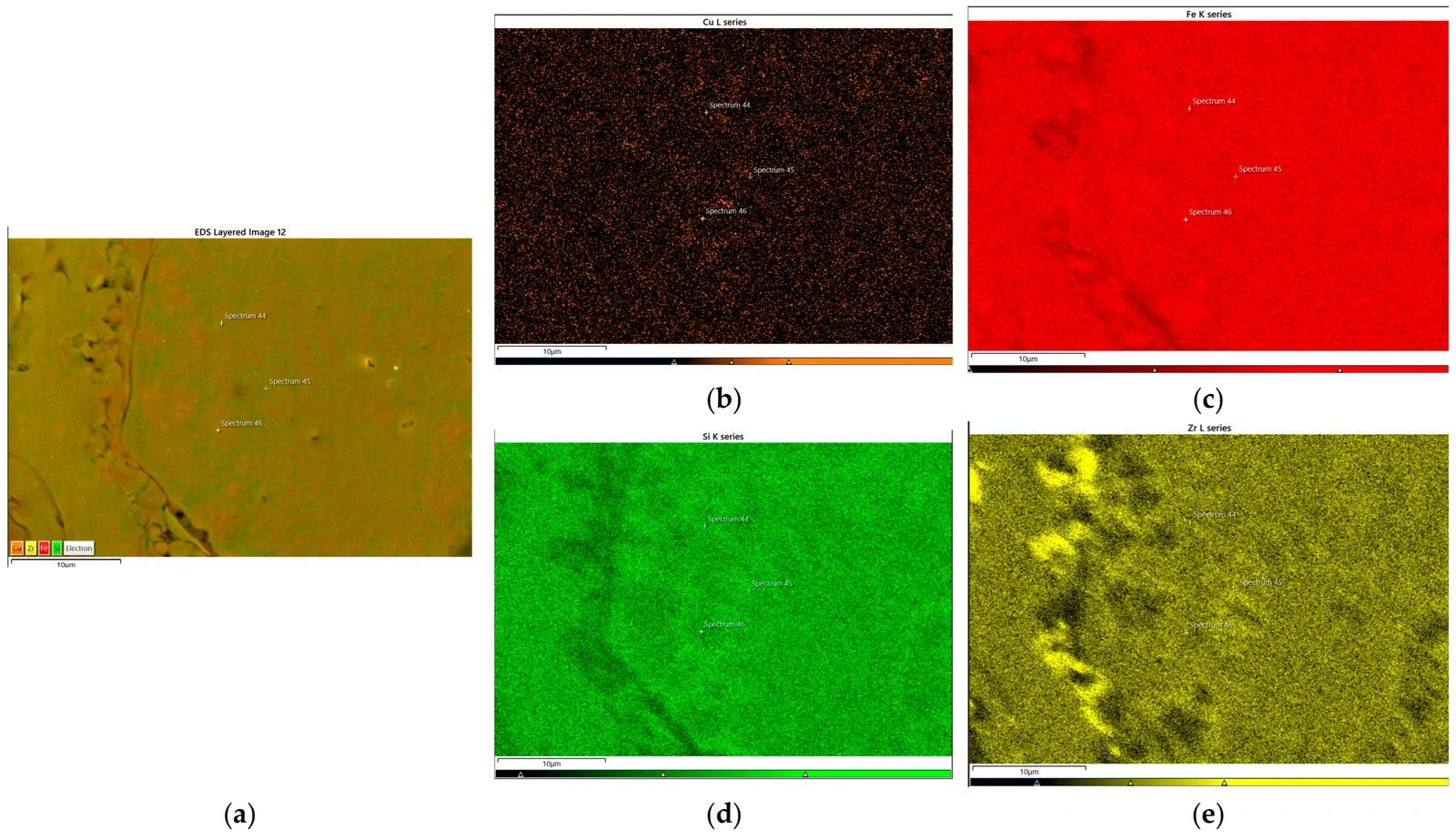
EDS mapping for PS90 sample: (**a**) reference image, (**b**) Cu, (**c**) Fe, (**d**) Si, (**e**) Zr.

Figure 5 presents the EDS elemental distribution maps of the PS90 coating. The Fe and Si maps show relatively uniform signal distributions across the analysed area, whereas the Zr map exhibits a distinctly heterogeneous pattern. In particular, regions of increased Zr signal intensity appear to be concentrated mainly along the boundaries and junctions between individual splats. This distribution may reflect local compositional variations associated with the splat interfaces; however, the possibility of topographical effects influencing the EDS signal cannot be excluded. The observed pattern should therefore not be interpreted as unequivocal evidence of Zr enrichment at these interfaces.

The Cu map also shows a relatively uniform distribution across the analysed region, without clearly distinguishable areas of pronounced local accumulation. The reference image reveals surface irregularities, including visible features associated with the splat boundaries. The correspondence between these morphological features and the Zr distribution suggests that the splat interfaces may be relevant to the local compositional characteristics of the coating. Nevertheless, further analysis would be required to establish whether the observed variations reflect actual differences in elemental concentration or are partly attributable to surface topography.

Overall, the EDS maps indicate a relatively uniform distribution of Fe, Si and Cu, accompanied by a heterogeneous Zr signal that appears to follow the splat boundaries and their junctions.

The EDS analysis of the PS90 coating shown in Table 3 confirmed the presence of all four alloying elements, including Cu. The measured Cu concentration was 1.07 at.%, which is close to the nominal composition of 1 at.%. EDS mapping was additionally used to examine the spatial distribution of the constituent elements within the analysed area. Nevertheless, the presence of Cu detected by EDS does not, by itself, establish whether Cu is incorporated into the existing phases or occurs in a separate Cu-rich phase.

Cross-sectional SEM observations confirmed the characteristic lamellar morphology typical of APS coatings. Local pores, interlamellar discontinuities, and crack-like defects were observed in all coatings, although their frequency depended on spray distance. Similar porosity levels were previously reported for Fe-based APS coatings. In [18], Fe_48_Mo_14_Cr_15_Y_2_C_15_B_6_ coatings exhibited porosity of approximately 2.9%, despite the use of substantially larger spray distances combined with higher plasma power, which likely compensated for particle cooling and improved melting behaviour.

Compared with HVOF spraying, APS generally involves lower particle velocities and longer residence times within the plasma plume [19,20], which promotes oxidation during flight and deposition. Oxide films formed at splat boundaries may weaken interlamellar bonding and contribute to pore formation. Similar effects of thermal input on coating densification were reported in [21], where increasing plasma power reduced porosity in Fe_73_Cr_2_Si_11_B_11_C_3_ coatings due to improved particle melting and splat cohesion.

The obtained results indicate that spray distance strongly influences particle thermal history and consequently the development of coating microstructure in the Fe-Zr-Si-Cu system. Although APS enabled deposition of continuous coatings with relatively low porosity, the increase in porosity observed at 100 mm suggests a relatively narrow processing window for this alloy under APS conditions.

### 3.4. Tribological Measurements

**Figure 6:**
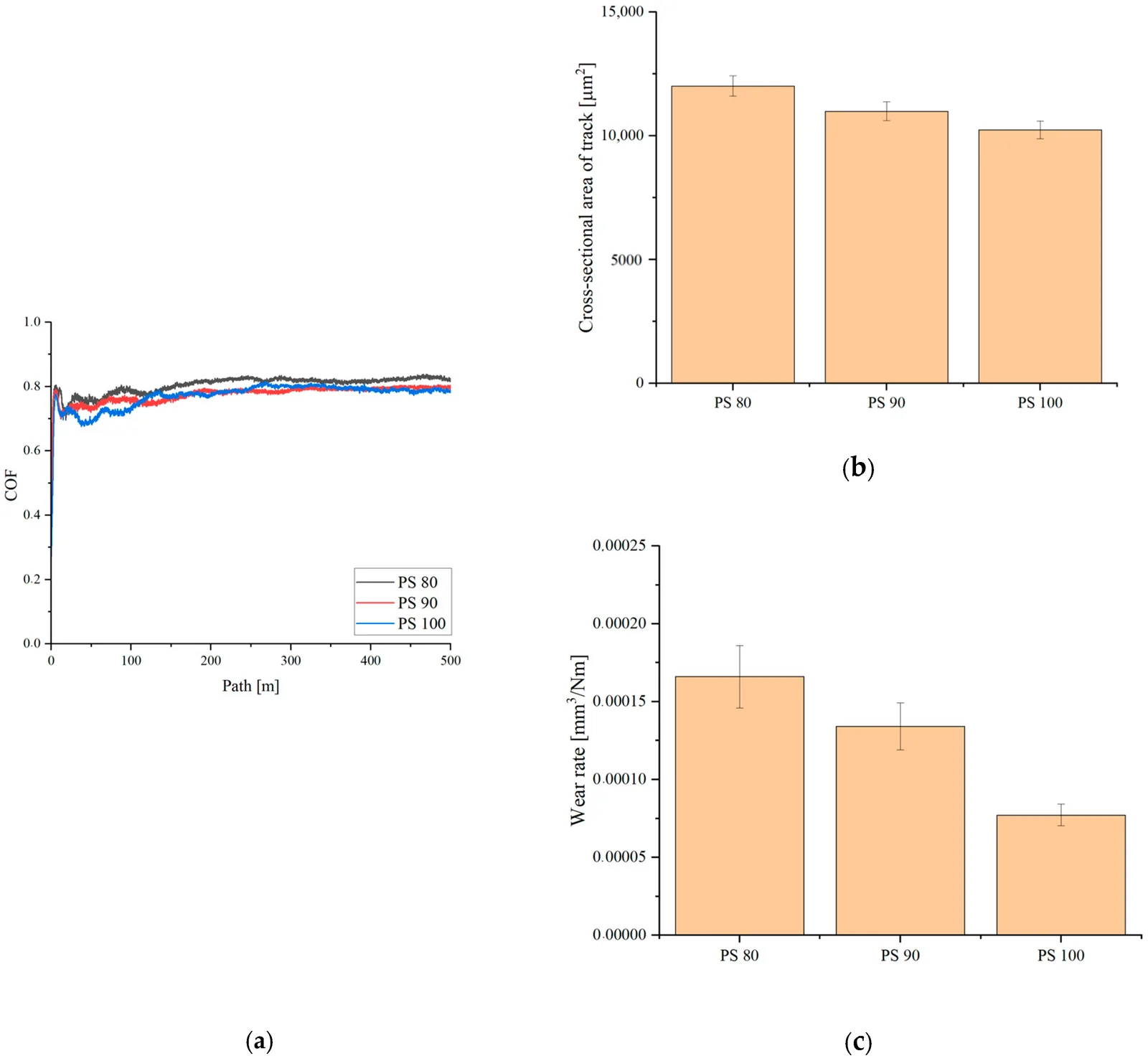
Tribological behaviour of APS coatings deposited at different spray distances: (**a**) COF as a function of sliding distance, (**b**) cross-sectional area of wear tracks, and (**c**) wear rate.

Figure 6 presents the tribological behaviour of APS coatings deposited at different spray distances under a normal load of 10 N. In all cases, the coefficient of friction (COF) curves exhibited a short running-in stage followed by relatively stable sliding conditions. The average COF slightly decreased with increasing spray distance, from 0.81 for PS80 to 0.78 and 0.77 for PS90 and PS100, respectively.

The wear performance of the coatings was evaluated based on the cross-sectional area of the wear tracks (Figure 5b) and the calculated wear rates (Figure 5c). Both parameters decreased with increasing spray distance, which is shown in Table 4. The PS80 coating exhibited the largest wear track area (~12,012 μm^2^) and the highest wear rate (1.66 × 10^−4^ mm^3^/N·m), whereas PS100 showed the smallest wear track area (~10,241 μm^2^) and the lowest wear rate (7.72 × 10^−5^ mm^3^/N·m). The relatively low standard deviations obtained or all measurements indicate good repeatability of the tribological tests.

The improved wear resistance of the PS100 coating is noteworthy because this sample also exhibited the highest porosity among the investigated coatings. However, PS100 simultaneously showed the highest hardness, suggesting that resistance to plastic deformation had a stronger influence on wear behaviour than porosity alone. The obtained results therefore indicate that the tribological response of the coatings was governed by a combination of hardness, splat cohesion, and local stress distribution within the lamellar microstructure.

Cross-sectional observations confirmed the characteristic lamellar morphology of APS coatings, containing interlamellar boundaries, pores, and partially unmelted particles that may locally concentrate stresses during sliding contact and promote crack initiation or material pull-out. Variations in splat solidification behaviour associated with spray distance likely contributed to the observed differences in wear resistance.

Similar tribological behaviour has previously been investigated for Fe-based thermal spray coatings [22,23,24]. In [22], tribological tests were performed under a normal load of 25 N and a sliding distance of 250 m, with an average coefficient of friction (COF) of approximately 0.3 reported for the investigated coatings. The reported wear-track cross-sectional areas were approximately 5000 µm^2^ for the laser-cladded coating and 10,000 µm^2^ for the plasma-sprayed coating. The latter value is comparable to that obtained for the plasma-sprayed coatings investigated in the present study, particularly at a stand-off distance of 100 mm. The smaller wear-track cross-sectional area reported for the laser-cladded coating was attributed to its denser microstructure and lower porosity. However, differences in processing methods and the resulting coating characteristics should also be considered when comparing wear performance. The COF measured in the present study was higher than the value reported in [22], although direct comparison is limited by differences in the alloy compositions and potentially in the tribological test conditions.

In [23], a different Fe-based alloy system was tested at room temperature (RT) and 600 °C. The average COF decreased from approximately 0.9 at RT to 0.7 at 600 °C. The authors also reported a wear rate of 1.67 ± 0.3 × 10^−5^ mm^3^/(N·m) at RT. For the coating deposited at a stand-off distance of 100 mm in the present study, the wear rate was 7.72 × 10^−5^ mm^3^/(N·m), approximately 4.6 times higher than the value reported in [23]. Nevertheless, both values are of the same order of magnitude. These differences should be interpreted in the context of the distinct alloy compositions, coating microstructures and test conditions. In particular, the Fe-Zr-Si-Cu alloy investigated here was developed primarily for soft magnetic applications, whereas differences in composition and phase constitution may affect its tribological response. However, the available results do not allow the higher COF and wear rate to be attributed to any single compositional or microstructural factor.

A different relationship between stand-off distance and COF was reported in [24] for APS coatings based on a Fe-C-P-Si-B-Mn-Cr-Mo alloy. The COF ranged from 0.22 to 0.65 under a normal load of 10 N and a test duration of 3600 s. The lowest value, 0.22, was obtained using a stand-off distance of 75 mm, whereas a value of 0.65 was reported at 100 mm for the corresponding parameter variants. Thus, the COF increased with increasing stand-off distance in that study. In contrast, the present results showed a decrease in COF with increasing stand-off distance, accompanied by reductions in wear-track area and wear rate. This opposite trend suggests that the influence of stand-off distance on tribological performance depends on the alloy system and the coating characteristics resulting from the selected spraying conditions. Consequently, the effect of stand-off distance cannot be generalised independently of the material composition, processing parameters and resulting microstructure.

### 3.5. Hardness Measurements

The Vickers microhardness results for the APS coatings are presented in Table 5. All deposited coatings exhibited substantially higher hardness than the AISI 304 stainless steel substrate, confirming formation of mechanically strengthened surface layers after plasma spraying. The substrate hardness was approximately 269.5 HV, whereas coating hardness ranged from 848.9 HV for PS80 to 903 HV for PS100.

A gradual increase in hardness with increasing spray distance was observed. The PS100 coating exhibited the highest hardness despite also showing the highest porosity among the investigated samples. Standard deviation values remained relatively low for all coatings, indicating good repeatability of the measurements, although slightly larger scatter was observed for PS100, likely due to its less homogeneous microstructure and higher defect population identified by SEM analysis.

The high hardness of the investigated coatings is associated with the crystalline multiphase structure identified by XRD. The presence of α-Fe(Si) together with Fe-Zr intermetallic phases likely increased resistance to localized plastic deformation, while rapid solidification during splat formation may additionally have contributed to microstructural refinement and strengthening of the deposited layers.

Similar hardness levels have previously been reported for Fe-based coatings produced using thermal spraying techniques [25,26,27,28]. Wu et al. [25] reported a microhardness of 935.93 ± 101.77 HV for an as-sprayed Fe_48_Cr_23_Mo_10_C_14_B_5_ coating produced by plasma spraying, measured under a load of 100 gf. The authors also investigated in situ reinforcement with Ti powder, which further increased the hardness. Zhong et al. [26] reported hardness values ranging from 1057.4 ± 2.4 to 1180.1 ± 2.3 HV for FeCrMoSi coatings. The coatings contained 13.38–17.17 wt.% Mo according to EDS analysis, and their high hardness may be associated with the alloy composition and resulting microstructure. The influence of Mo content on the hardness of Fe-based alloys has also been discussed in [27]. In addition, Xie and Wang [28] reported a hardness of approximately 900 HV for a detonation-sprayed FeCrMoCB coating deposited on a 304 stainless steel substrate, while a lower value of approximately 580 HV was measured in the coating–substrate interface region.

In comparison, the coatings investigated in the present study exhibited mean hardness values ranging from 848.9 ± 22.1 to 903.0 ± 35.8 HV_0.1_. These values are within the range reported for other Fe-based thermally sprayed coatings, although they are generally lower than those measured for some Mo-, Cr- and B-containing systems. This difference may be related to variations in alloy composition, phase constitution and coating microstructure. The Fe-Zr-Si-Cu alloy investigated here was primarily developed for soft magnetic applications rather than specifically optimised for high hardness or wear resistance. Nevertheless, differences in deposition methods, indentation loads and testing conditions should be considered when comparing results across different studies.

Among the coatings investigated in the present study, PS100 exhibited the highest mean hardness (903.0 ± 35.8 HV_0.1_), significantly exceeding the values measured for PS80 and PS90. However, PS100 also exhibited higher porosity than the other coatings. Therefore, its higher hardness cannot be explained by reduced porosity alone. Differences in phase distribution, local microstructure and splat solidification behaviour may have contributed to the observed trend, although their individual effects cannot be established conclusively from the available results.

### 3.6. Electrochemical Measurements

**Figure 7:**
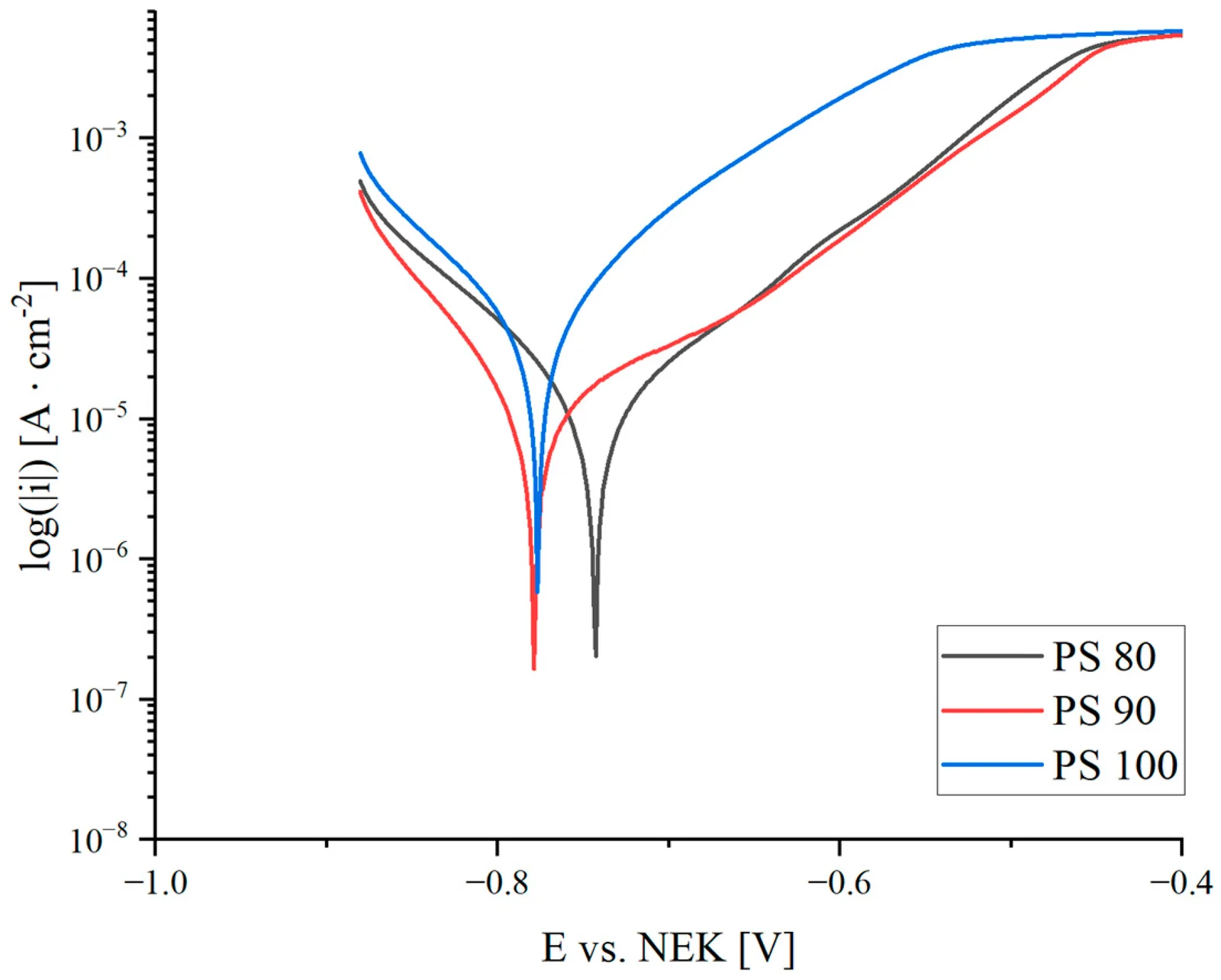
Polarization curves in 3.5 wt.% NaCl solution after 1 h of immersion.

The electrochemical behaviour of the coatings deposited at different spray distances was evaluated in 3.5 wt.% NaCl solution. Potentiodynamic polarization results are presented in Figure 7, while the electrochemical parameters derived from the polarization measurements are summarized in Table 6.

All coatings exhibited an initial rapid change in open-circuit potential followed by gradual stabilization during immersion. The PS100 coating showed the least negative OCP values, whereas PS90 exhibited the most negative potential response. Despite these differences in corrosion potential, the polarization measurements revealed more pronounced differences in corrosion kinetics.

Among the investigated samples, the PS90 coating exhibited the lowest corrosion current density (j_corr_ = 2.92 μA/cm^2^) together with the lowest corrosion rate (0.03 mm/year), indicating the most favourable corrosion performance. PS80 coating showed a somewhat higher corrosion current density (4.26 μA/cm^2^) and a slightly higher corrosion rate (0.05 mm/year). In contrast, PS100 showed substantially higher corrosion activity, with j_corr_ increasing to 11.21 μA/cm^2^ and polarization resistance decreasing to 0.56 kΩ·cm^2^. Although this coating exhibited a relatively noble corrosion potential (E_corr_ = −0.71 V), the high corrosion current density indicates accelerated electrochemical degradation.

**Figure 8:**
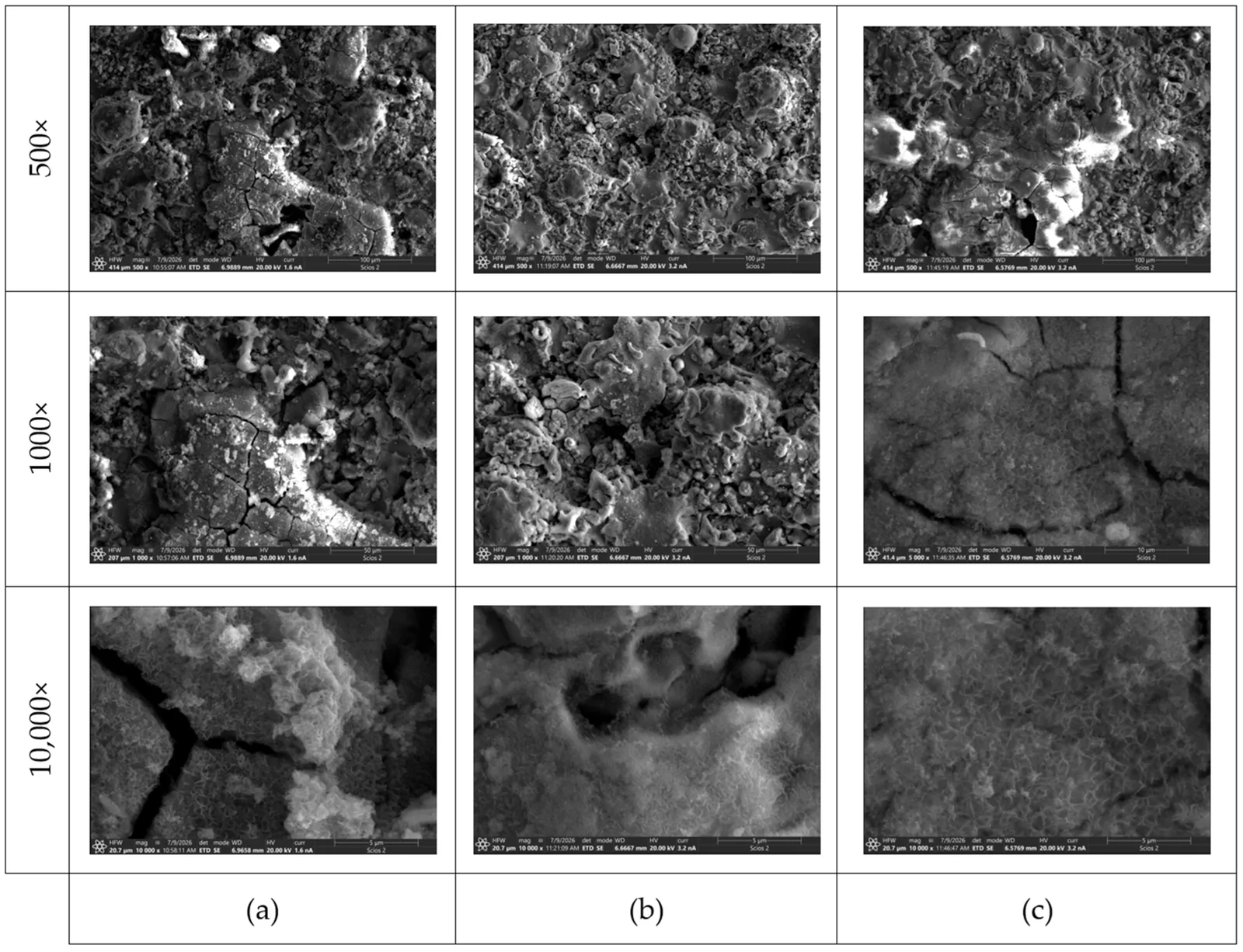
SEM imaging after electrochemical tests for spraying distance of: (**a**) 80 mm, (**b**) 90 mm, (**c**) 100 mm.

Figure 8 presents SEM images of the coating surfaces after electrochemical testing. The observed morphology revealed distinct differences in the extent and character of surface degradation. The PS80 coating exhibited pronounced local degradation, with irregular surface features and extensive cracking of the affected regions. The PS90 surface was also heterogeneous and covered by corrosion-affected structures but showed less pronounced large-scale cracking than PS80 and PS100. In contrast, the PS100 coating exhibited large, clearly defined degraded regions with a pronounced network of surface cracks, particularly evident at 500× and 1000× magnification. At higher magnification, all coatings exhibited fine-scale surface structures associated with corrosion-induced modification and the formation or accumulation of corrosion products.

The pronounced surface cracking and degradation observed for PS100 are consistent with its electrochemical response, characterized by the highest jcorr and the lowest R_p_. This behaviour can be related to the higher porosity and greater population of interlamellar discontinuities previously observed in this coating. Such defects may facilitate electrolyte penetration into the coating and provide preferential pathways for localized corrosion. In APS coatings, pores, splat boundaries, and microcracks can act as active sites for corrosion initiation, particularly in chloride-containing environments.

**Figure 9:**
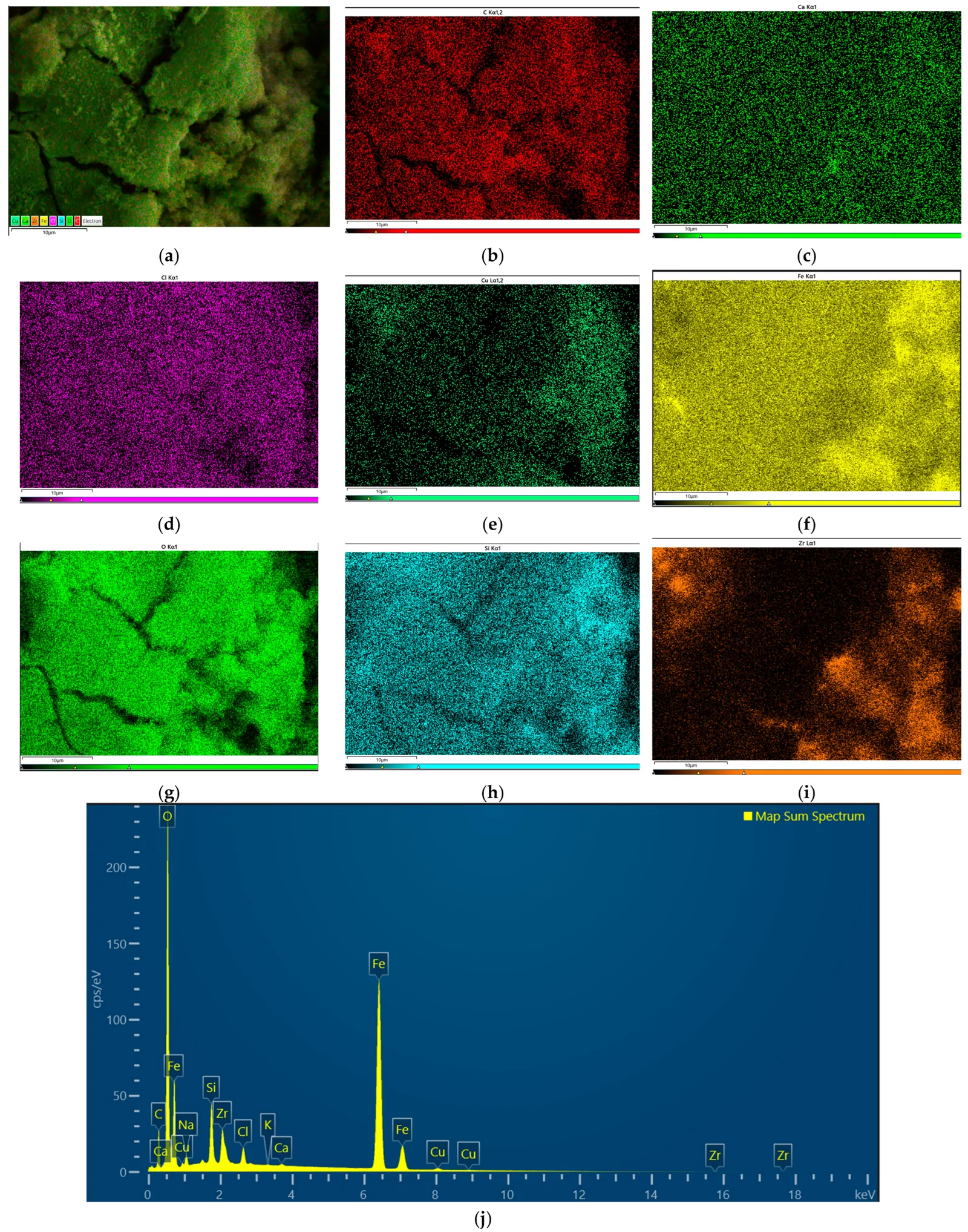
EDS mapping for PS80 sample: (**a**) reference image, (**b**) C, (**c**) Ca, (**d**) Cl, (**e**) Cu, (**f**) Fe, (**g**) O, (**h**) Si, (**i**) Zr, (**j**) spectrum map.

Figure 9 shows the EDS mapping after corrosion testing. The oxygen signal is particularly prominent, suggesting the presence of oxygen-containing surface products, potentially including metal oxides. Notable is that expect Zr all main elements of the alloy are evenly distributed. The Fe, Si and Cu signals appear relatively uniformly distributed across the analysed area, whereas the Zr map exhibits local variations in signal intensity, which may be associated with surface topography or local compositional heterogeneity. Also Zr is more prone to embedded into cracks, while expect Fe, rest of elements stayed mostly outside.

Signals corresponding to Na and Cl were also detected. Their presence can be attributed to the NaCl solution used as the corrosive medium, although residual electrolyte on the surface cannot be distinguished from species incorporated into corrosion products based on EDS alone. A small amount of Ca was also detected; however, its origin remains unclear and cannot be conclusively determined from the available data. There is a possibility that this may be precipitation from reagents like NaCl solution or distilled water used to clean samples.

The EDS analysis additionally revealed a relatively high carbon content. One possible source is the carbon adhesive tape used to mount the specimen for SEM examination. However, given the low energy of the characteristic X-ray emission of carbon, its detection and quantitative determination by EDS may be affected by the detector characteristics and measurement conditions. Alternatively, the carbon signal may be associated with carbon-containing surface contaminants or species formed during reactions occurring in the corrosive environment. Its origin cannot be established unambiguously from the present results.

Overall, the EDS analysis confirms the presence of oxygen-containing surface products after corrosion exposure. Nevertheless, further investigation would be required to determine their chemical composition and establish the origin of the detected carbon and calcium.

**Figure 10:**
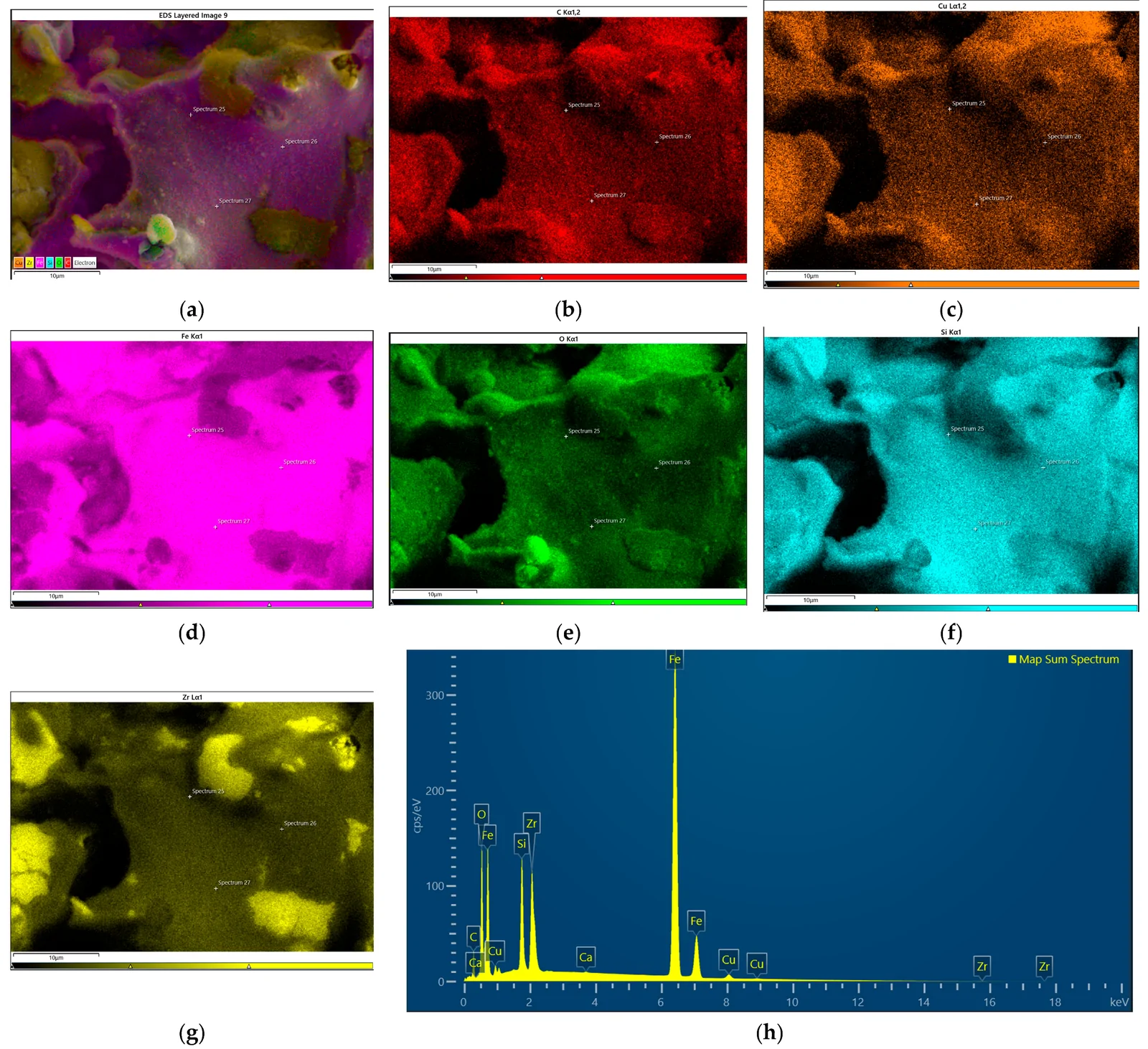
EDS mapping for PS90 sample: (**a**) reference image, (**b**) C, (**c**) Cu, (**d**) Fe, (**e**) O, (**f**) Si, (**g**) Zr, (**h**) spectrum map.

Figure 10 presents the EDS elemental maps of the PS90 coating after corrosion testing. The elemental maps show a relatively uniform distribution of Fe, Cu and Si across the analysed area, whereas the Zr map exhibits noticeable local variations in signal intensity. The oxygen map reveals differences in signal intensity between individual surface regions, suggesting local variations in the distribution of oxygen-containing species. These variations appear to correspond partly to the heterogeneous surface morphology observed in the reference image. The EDS spectrum confirms the presence of the main alloying elements, namely Fe, Zr, Si and Cu, as well as oxygen. The presence of oxygen is consistent with the formation of oxygen-containing surface products during exposure to the corrosive environment, potentially including metal oxides. However, EDS analysis alone does not allow their chemical composition to be identified unambiguously. Compared with the PS80 sample, no distinct Na or Cl signals are labelled in the presented spectrum of PS90. This difference may reflect variations in the presence of residual electrolyte or surface species, although the absence of labelled peaks does not necessarily indicate that these elements are entirely absent. Overall, the results indicate a heterogeneous distribution of oxygen-containing species on the PS90 surface after corrosion testing.

**Figure 11:**
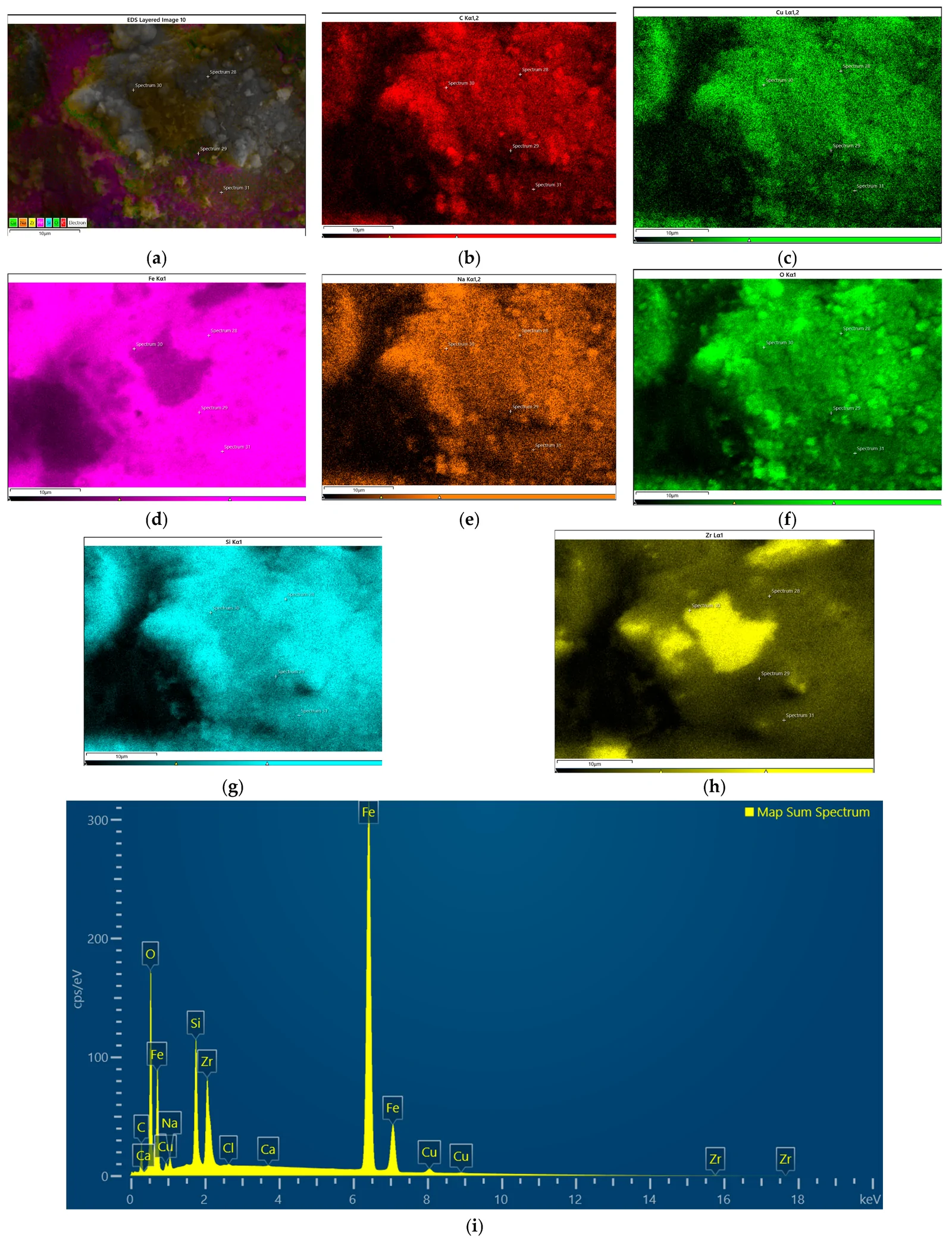
EDS mapping for PS100 sample: (**a**) reference image, (**b**) C, (**c**) Cu, (**d**) Fe, (**e**) Na, (**f**) O, (**g**) Si, (**h**) Zr, (**i**) spectrum map.

Figure 11 presents the EDS elemental distribution maps of the PS100 coating after corrosion testing in a 3.5 wt.% NaCl solution. The elemental maps reveal a heterogeneous surface morphology accompanied by noticeable variations in the spatial distribution of the detected elements. Iron and copper are distributed across the analysed area, although local differences in signal intensity can be observed. In contrast, the Si and Zr maps exhibit more pronounced variations, with regions of increased signal intensity, suggesting local compositional heterogeneity within the coating.

The oxygen map reveals a non-uniform distribution, with locally intensified signals that may indicate the accumulation of oxygen-containing corrosion products, potentially including metal oxides and hydroxides. The EDS analysis revealed an oxygen content of 29.36 at.%, which was lower than that observed for PS80 (49.56 at.%) but higher than that for PS90 (20.95 at.%). Additionally, sodium (1.43 at.%) and a small amount of chlorine (0.06 at.%) were detected, which may be associated with residual electrolyte originating from the NaCl solution used during corrosion testing.

Carbon was also detected (21.08 at.%), potentially originating from the carbon adhesive tape used for specimen mounting or surface contamination. However, due to the relatively low energy of its characteristic X-ray emission, the quantitative determination of carbon by EDS is subject to additional uncertainty. Trace amounts of calcium (0.04 at.%) were also identified, although their origin remains unclear.

Among the investigated coatings, PS100 exhibited the highest corrosion current density (11.21 μA/cm^2^) and the lowest polarization resistance (0.56 kΩ·cm^2^), indicating the least favourable electrochemical corrosion behaviour under the investigated conditions. The heterogeneous distribution of oxygen and alloying elements observed in the EDS maps is consistent with spatial variations in the surface condition after corrosion exposure. However, the EDS results alone do not establish a direct relationship between the observed elemental distributions and the electrochemical corrosion resistance.

The measured iron content also varied considerably between the samples, reaching 20.81 at.% for PS80, 54.68 at.% for PS90 and 38.64 at.% for PS100. The higher iron content and lower oxygen content observed for PS90 indicate that the analysed region contained a comparatively larger proportion of Fe relative to oxygen than the corresponding regions of PS80 and PS100. However, these differences cannot be directly interpreted as differences in the amount of iron dissolved during corrosion, since the EDS results also reflect the composition of the surface products and the normalisation of the detected elements.

Differences were also observed in the measured contents of Si, Zr and Cu. The Si content ranged from 2.75 at.% for PS80 to 6.98 at.% for PS90, while the corresponding Zr contents were 1.03 and 2.39 at.%, respectively. The Cu content varied between 0.32 at.% for PS80 and 0.95 at.% for PS90. These values differ from the nominal alloy composition, although the observed differences alone do not establish selective dissolution or enrichment of individual alloying elements.

Carbon was detected in all three samples, with contents of 23.26 at.% for PS80, 14.02 at.% for PS90 and 21.08 at.% for PS100. Since carbon is not part of the nominal alloy composition, its origin remains uncertain. Possible sources include contamination during specimen preparation, the carbon adhesive tape used for mounting, or carbon-containing surface species. Its contribution to the EDS results should therefore be considered when interpreting the normalised concentrations of the other elements.

Sodium was detected in PS80 (1.46 at.%) and PS100 (1.43 at.%), whereas chlorine was detected in PS80 (0.70 at.%) and PS100 (0.06 at.%). These elements may originate from residual electrolyte associated with the NaCl corrosion medium; however, their detection does not establish whether they are present as residual salts or incorporated into corrosion products. Small amounts of calcium were detected in all samples (0.03–0.08 at.%), while potassium was detected only in PS80 (0.02 at.%). The origins of these trace signals could not be established from the available EDS data.

Overall, the EDS results demonstrate differences in the elemental composition of the analysed surface regions following corrosion testing. The high oxygen contents are consistent with the presence of oxygen-containing surface products, while the variations in the measured concentrations of the alloying elements indicate differences in the local surface composition. Nevertheless, the EDS results alone do not allow the chemical nature of the corrosion products or the mechanisms responsible for the observed compositional differences to be determined conclusively.

The electrochemical behaviour observed in the present study is comparable to previously reported results for APS Fe-based coatings [24,29]. In [24], Fe-Cr-Si-B-C coatings were deposited using different plasma power levels. Since all coatings in the present study were produced at a constant plasma power of 19.3 kW, the coating deposited at 18.6 kW in [24] provides a useful reference for comparison. This coating exhibited a corrosion current density of 18 µA/cm^2^ and a corrosion potential of −0.692 V. The corresponding j_corr_ values for PS80 and PS90 were 18.24 and 19.25 µA/cm^2^, respectively, indicating similar corrosion current densities under the investigated conditions. Their corrosion potentials were less negative, at −0.58 and −0.59 V, respectively. However, the differences in E_corr_ alone do not establish superior corrosion resistance, and the electrochemical performance should be assessed by considering the corrosion current density alongside the corrosion potential and other available electrochemical parameters.

In contrast, PS100 exhibited a higher j_corr_ of 28.81 µA/cm^2^, indicating a higher corrosion rate under the conditions of the electrochemical test, assuming comparable measurement and data-analysis procedures. This increase may be associated with differences in the coating microstructure, including porosity and defects that can facilitate electrolyte penetration. Nevertheless, the available results do not establish a direct causal relationship between the higher current density and any individual microstructural feature.

A dependence of electrochemical behaviour on stand-off distance was also reported in [29] for APS Fe-based coatings. The authors observed lower corrosion-related parameter values at a stand-off distance of 75 mm and discussed the importance of adjusting multiple processing parameters to obtain favourable electrochemical performance. In the present study, the higher j_corr_ measured for PS100 indicates that the longest investigated stand-off distance did not provide the best electrochemical performance. However, the effect of stand-off distance should be interpreted in conjunction with the resulting coating characteristics and the other processing conditions rather than as a universal relationship.

Besides porosity effects, the multiphase crystalline structure identified by XRD may additionally contribute to the electrochemical response of the coatings. The coexistence of α-Fe(Si) and Fe-Zr intermetallic phases may promote local micro-galvanic interactions, particularly near splat boundaries and structurally heterogeneous regions, thereby accelerating localized corrosion after electrolyte penetration into the coating interior.

Results indicate that spray distance strongly influences corrosion behaviour through its effect on coating densification and microstructural integrity. Among the investigated coatings, PS90 exhibited the most favourable combination of low porosity and good electrochemical stability.

### 3.7. Relationship Between Structure and Properties of APS Coatings Made from Fe_79_Zr_6_Si_14_Cu_1_

Results from the present study demonstrate a strong relationship between APS processing conditions, coating microstructure, and functional properties. All deposited coatings exhibited predominantly crystalline multiphase structures composed of α-Fe(Si) together with Fe-Zr intermetallic phases, which contributed to substantially higher hardness compared with the stainless-steel substrate.

SEM observations revealed a characteristic lamellar microstructure containing intersplat boundaries, pores, and localized unmelted regions, which are commonly observed in plasma-sprayed coatings. Differences in coating porosity and microstructural cohesion were observed at different spray distances. These differences may be related to variations in particle thermal history and splat solidification behaviour. In particular, the longer flight distance may have promoted particle cooling prior to impact, potentially affecting particle melting and splat spreading and contributing to defect formation. However, these mechanisms were not directly measured in the present study.

The functional behaviour of the coatings was governed by the combined influence of phase constitution and defect population. Higher hardness contributed to improved wear resistance, whereas pores and interlamellar discontinuities strongly affected electrochemical behaviour. In particular, the PS100 coating exhibited the highest hardness together with the lowest wear rate, indicating enhanced resistance to material removal during sliding. However, its increased porosity also facilitated electrolyte penetration and accelerated corrosion processes.

Results therefore indicate a trade-off between mechanical and electrochemical performance. Larger spray distances improved hardness and wear resistance but simultaneously reduced corrosion resistance due to the increased defect population within the lamellar structure. Among the investigated deposition conditions, the coating produced at a spray distance of 90 mm exhibited the most favourable overall balance of properties. This behaviour was likely associated with more favourable thermal conditions during APS deposition, where sufficient particle melting was achieved without excessive in-flight cooling. Consequently, the PS90 coating developed a relatively cohesive microstructure with low porosity, resulting in favourable hardness, wear resistance, and corrosion behaviour simultaneously.

## 4. Conclusions

To the best of our knowledge, this is the first study to investigate the microstructure and functional properties of Fe_79_Zr_6_Si_14_Cu_1_ coatings deposited by atmospheric plasma spraying (APS). Based on the obtained results, the following conclusions can be drawn:

- APS processing of the investigated low-GFA Fe-Zr-Si-Cu alloy resulted in formation of predominantly crystalline coatings composed of α-Fe(Si) and Fe-Zr intermetallic phases. No detectable amorphous phase was identified despite the rapid solidification conditions associated with APS. This behavior was attributed to the limited glass-forming ability of the alloy together with splat-based solidification, repeated thermal cycling, and heterogeneous nucleation during coating build-up.
- All coatings exhibited a characteristic lamellar microstructure typical of plasma-sprayed materials. Spray distance strongly affected coating densification and defect population through its influence on particle thermal history and splat formation behavior. Increasing spray distance within the investigated range was associated with higher porosity.
- The functional properties of the coatings were closely related to their phase constitution and microstructural characteristics. Increasing spray distance resulted in higher hardness and improved wear resistance, with the PS100 coating exhibiting the lowest wear rate.
- Corrosion resistance deteriorated with increasing spray distance due to increased porosity and facilitated electrolyte penetration through interlamellar defects. Among the investigated coatings, PS90 exhibited the most favorable electrochemical performance.
- Considering the combined influence of microstructure, hardness, tribological measurements, and corrosion resistance, the coating deposited at a spray distance of 90 mm exhibited the most favorable overall balance of properties.

Future work should focus on post-processing treatments aimed at reducing coating porosity and modifying phase composition, as well as on a detailed investigation of oxide formation and thermodynamic phase stability in the Fe-Zr-Si-Cu system. In addition, alternative thermal spraying techniques characterized by higher particle velocities and lower oxidation tendencies may provide more favourable conditions for the formation of an amorphous fraction during deposition of low-GFA Fe-based alloys.

## Figures and Tables

**Table 1:** Nominal chemical composition of Fe_79_Zr_6_Si_14_Cu_1_ alloy.

Elements				
	Iron	Zirconium	Silicon	Copper
% at.	79	6	14	1

**Table 2:** Process parameters used for APS deposition in present study.

Parameter	Value
Plasma gas	Ar
Plasma gas flow	45 slpm
Plasma power	19.3 kW
Voltage	39 V
Powder feed rate	20 g/min
Powder carrier gas	Ar
Powder carrier gas flow	2.5 slpm
Preheating/interpass temperature	200 °C
Maximum substrate temperature	400 °C
Traverse speed	400 mm/s
Spraying distance	80, 90, 100 mm

**Table 3:** Elemental composition (at.%) of ASC, determined by EDS.

Element	Fe	Zr	Si	Cu	Rest
Nominal chemical composition, at. %	79	6	14	1	0
PS90, at. %	81.68	4.70	12.46	1.07	0.10

**Table 4:** Average COF, wear track area, and wear rate of APS coatings deposited at different spray distances.

Parameter	Average COF			Wear Area			Wear Rate		
Unit	μ			μm^2^			mm^3^/N·m		
Sample Denomination	PS 80	PS 90	PS 100	PS 80	PS 90	PS 100	PS 80	PS 90	PS 100
Mean	0.81	0.78	0.77	12,012	10,993	10,241	1.66 × 10^−4^	1.34 × 10^−4^	7.72 × 10^−5^
Standard deviation	0.03	0.03	0.04	407	389	356	0.1 × 10^−4^		0.5 × 10^−5^
Coefficient of variation [%]	2.9	3.8	5.2	3.9	3.7	4	6	7.4	6.5

**Table 5:** Average values of coatings microhardness.

Parameter	Average Hardness		
Unit	HV_0.1_		
Sample Denomination	PS 80	PS 90	PS 100
Mean	848.9	865.3	903
Standard deviation	22.1	27.4	35.8
Coefficient of variation [%]	2.6	3.2	4

**Table 6:** Electrochemical parameters of the coatings after 1 h immersion in 3.5 wt.% NaCl solution.

Parameter	SD	β_a_	β_c_	E_ocp_	E_corr_	R_p_	j_corr_	v_corr_
Unit	mm	mV/dec	mV/dec	V	V	KΩ·cm^2^	μA/cm^2^	mm/year
	80	38.27	48.76	−0.58	−0.74	2.19	4.26	0.05
	90	23.99	35.87	−0.59	−0.79	2.14	2.92	0.03
	100	32.28	26.09	−0.51	−0.71	0.56	11.21	0.13

## Data Availability

The original contributions presented in the study are included in the article. Further inquiries can be directed to the corresponding author.

## Abbreviations

The following abbreviations are used in this manuscript:

APS	Atmospheric Plasma Spraying
BSE	Backscattered Electrons
GFA	Glass-Forming Ability
XRD	X-ray diffraction
SEM	Scanning Electron Microscope
COF	Coefficient of Friction
RT	Room Temperature
SD	Spraying Distance
TS	Thermal Spraying
